# Synthesis of SMT022357 enantiomers and *in vivo* evaluation in a Duchenne muscular dystrophy mouse model

**DOI:** 10.1016/j.tet.2019.130819

**Published:** 2020-01-10

**Authors:** Arran Babbs, Adam Berg, Maria Chatzopoulou, Kay E. Davies, Stephen G. Davies, Benjamin Edwards, David J. Elsey, Enrico Emer, Aude L.A. Figuccia, Ai M. Fletcher, Simon Guiraud, Shawn Harriman, Lee Moir, Neil Robinson, Jessica A. Rowley, Angela J. Russell, Sarah E. Squire, James E. Thomson, Jonathon M. Tinsley, Francis X. Wilson, Graham M. Wynne

**Affiliations:** a Department of Anatomy and Genetics, MDUK Oxford Neuromuscular Centre, University of Oxford, Oxford OX1 3PT, UK; b Department of Chemistry, University of Oxford, Chemistry Research Laboratory, Mansfield Road, Oxford, OX1 3TA, UK; c Summit Therapeutics plc, 136a Eastern Avenue, Milton Park, Abingdon, OX14 4SB, UK; d S.H.B. Enterprises Ltd, 55 Station Road, Beaconsfield, HP19 1QL, UK; e Department of Pharmacology, University of Oxford, Mansfield Road, Oxford, OX1 3PQ, UK

**Keywords:** Duchenne muscular dystrophy, Utrophin modulator, Phosphinate, Enantioselective synthesis, Preclinical candidate

## Abstract

Following on from ezutromid, the first-in-class benzoxazole utrophin modulator that progressed to Phase 2 clinical trials for the treatment of Duchenne muscular dystrophy, a new chemotype was designed to optimise its physicochemical and ADME profile. Herein we report the synthesis of SMT022357, a second generation utrophin modulator preclinical candidate, and an asymmetric synthesis of its constituent enantiomers. The pharmacological properties of both enantiomers were evaluated *in vitro* and *in vivo*. No significant difference in the activity or efficacy was observed between the two enantiomers; activity was found to be comparable to the racemic mixture.

## Highlights

• A stereoselective synthesis was devised to access enantiomers of phosphinate SMT022357.
• SMT022357 enantiomers have an improved ADME profile compared to ezutromid.
• SMT022357 enantiomers show comparable activity and improve the dystrophic phenotype in *mdx* mice.

## Graphical abstract

**Figure:**
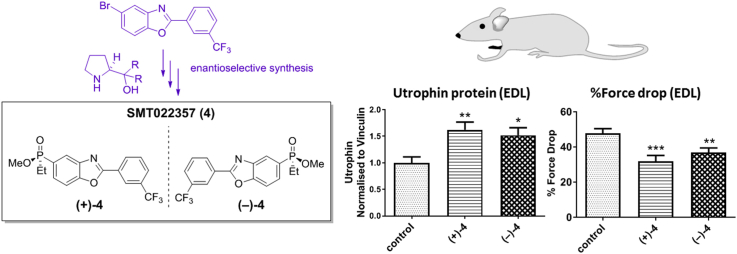

## 1 Introduction

Duchenne muscular dystrophy (DMD) is an X linked, recessive, fatal, muscle-wasting disease [1]. This disorder affects 1 in 5000 boys and its incidence is continuing to rise in all populations worldwide [2]. Dystrophin is essential for muscle membrane stability as it acts as a linker between the internal cytoskeleton of the muscle cell and the extracellular matrix to form the dystrophin-associated protein complex (DAPC) at the sarcolemma [3]. DMD is caused by mutations in the DMD gene leading to the expression of non-functioning or truncated dystrophin. This in turn gives rise to a less stable DAPC and progressive muscle degeneration. Currently there is no cure for all DMD patients and treatments aim to lessen the symptoms of the disease without addressing the cause. Initial symptoms appear when patients are young children and loss of ambulation occurs in the early teens. Due to the wide-reaching implications of the disease on the human body, the condition usually results in death for patients in their twenties or thirties due to heart or respiratory failure [4,5].

Amongst the strategies being pursued to treat DMD, such as exon-skipping therapy [6] and viral gene therapy [7], the upregulation of utrophin production could provide a long-term treatment for all patients with DMD. In mouse and dog models of the disease, it has been found that the lack of dystrophin could be compensated for by the increased production of the related protein utrophin. Therefore, the administration of small molecules which transcriptionally upregulate the production of utrophin could be an effective treatment for DMD [8].

Utrophin is structurally similar to dystrophin and is able to provide stability to muscle cells in a similar role to dystrophin but at different stages of fibre maturity. This has the advantage over the other treatments in development in that it should be successful in treating all patients, regardless of the genetic mutation responsible for their illness [9], and it does not require immunosuppression [7].

**Fig. 1:**
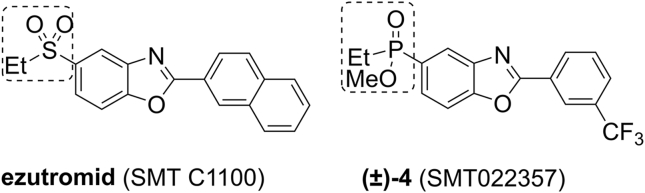
Chemical structures of ezutromid and SMT022357 ((±)-**4**). Highlighted is the position of bioisosteric replacement.

In this context, ezutromid (Fig. 1), developed by Summit Therapeutics plc, has been the first orally bioavailable small molecule specifically designed to target the utrophin-A promoter. Unfortunately, despite the encouraging interim 24-week data where evidence of target engagement were observed (reduced muscle fibre damage in combination with an increase in utrophin protein), ezutromid was discontinued in June 2018 after primary and secondary endpoints were missed at the conclusion of a Phase 2 trial [10].

In parallel to ezutromid’s clinical evaluation, second generation utrophin modulators were also being developed. This new compound series arose from bioisosteric replacement of the sulfone moiety with a phosphinate ester and conferred improved physicochemical properties and a more stable metabolic profile [8]. In particular SMT022357 was found to exhibit *in vivo* efficacy in the *mdx* mouse (2-week-old mice, orally dosed daily with 30 mg/kg SMT022357 or vehicle for 5 weeks) leading to increased utrophin expression in skeletal and cardiac muscles [8].

Herein, we report the synthesis of SMT022357, its resolution into, and *de novo* enantioselective synthesis of, its constituent enantiomers and the preliminary results of their *in vivo* efficacy.

## 2 Results and discussion

### 2.1 Chemistry

**Scheme 1:**
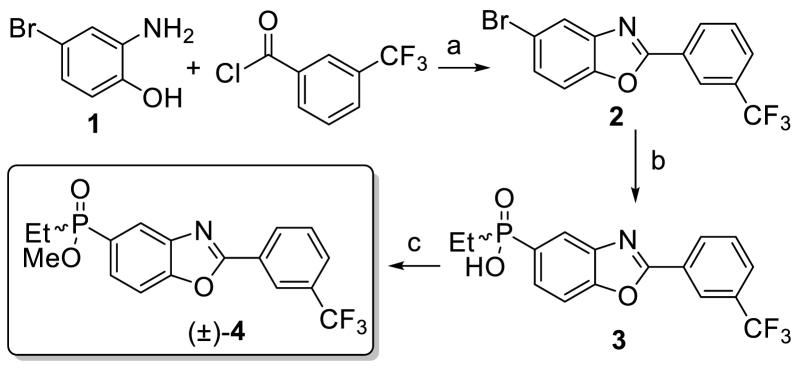
Synthesis of SMT022357 ((±)-**4**). Reagents and conditions: (a) CH_3_SO_3_H, toluene, 55 °C, 8 h, 73%; (b) Pd(OAc)_2_, EtP = (O)(OH), Xantphos, DIPEA, PhMe/DME, reflux, 2 h, 94%; (c) 1. (COCl)_2_, THF, 50 °C, 3 h; 2. NaOMe, rt, 1 h, 95%.

The optimised synthesis of (±)-**4** is illustrated in Scheme 1. Cyclocondensation of the commercially available 2-amino-4-bromophenol **1** and 3-(trifluoromethyl)benzoyl chloride under acidic conditions at 55 °C, afforded benzoxazole **2** in 73% yield. Subsequent cross-coupling of aryl bromide **2** with 1.2 equivalents of ethylphosphinic acid, in the presence of 0.5 mol% of Pd(OAc)_2_/Xantphos and 1.5 equivalents of *N,N*-diisopropylethylamine (DIPEA) generated the corresponding benzoxazoylphosphinic acid **3** in almost quantitative yield (94%). Finally, treatment of **3** with oxalyl chloride in the presence of a catalytic amount of DMF and subsequent esterification with NaOMe delivered the corresponding methyl ethyl(2-(3-(trifluoromethyl)phenyl)-benzo[*d*]oxazol-5-yl)phosphinate (±)-**4** in 95% yield as a racemic mixture. Separation of the enantiomers obtained was successfully performed using preparative chiral HPLC to afford (+)-**4** and (−)-**4** in 47% and 45% yield, respectively (Supplementary data).

**Scheme 2:**
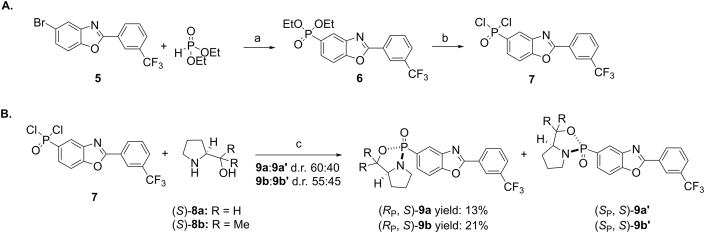
A. Synthesis of compound **7**. Reagents and conditions: (a) Pd(PPh_3_)_4_, Et_3_N, PhMe, reflux, 2 h, quant.; (b) SOCl_2_, DMF, reflux, 16 h, quant. B. Diastereoselective formation of oxazophospholidines **9a** and **9b** starting from phosphonic dichloride **7**. Reagents and conditions: (c) Et_3_N, THF, rt, 4 h.

In an attempt to produce significant quantities and to define the absolute configurations of the two enantiomers, an asymmetric route was used. Although several synthetic strategies have been developed in this field, one of the most common involves the use of chiral auxiliaries. Chiral pool precursors including ephedrine [11], menthol [12,13], and prolinol derivatives [[14], [15], [16]] have been used for inducing asymmetric induction to prochiral organophosphorus compounds. In our case, the absolute configuration of the *P*-stereocentre was determined by using enantiopure prolinols to afford the corresponding oxazaphospholidine oxide (Scheme 2). This method, first reported by Koizumi and co-workers, has been shown to be a simple but effective way to control selectivity in the *P*-centre [17]. Moreover, the same group has proved that the resulting cyclic oxazaphospholidine oxide reacts stereoselectively with different *C*- and *O*-nucleophiles, providing the corresponding phosphinates in enantioenriched form [18].

Coupling aryl bromide **5** with diethyl phosphite using Pd(PPh_3_)_4_/Et_3_N afforded compound **6** in quantitative yield (Scheme 2A), which was subsequently fully converted to (2-(3-(trifluoromethyl)phenyl)benzo[*d*]oxazol-5-yl)phosphonic dichloride **7** by treatment with a catalytic amount of DMF in SOCl_2_, at 70 °C. Notably, highly concentrated conditions (2.0 M) were required to fully displace the ethoxy groups.

Two chiral auxiliaries were used to explore potential improvements in crystallinity, stability, and/or higher levels of diastereoselectivity in the nucleophilic displacement reaction at P. As a result, compound **7** was reacted with either (*S*)-prolinol **8a** or α,α′-dimethyl-(*S*)-prolinol **8b** in the presence of triethylamine, in THF. The resulting cyclic oxazaphospholidine oxides **9a** and **9b** were obtained with diastereomeric ratios varying from 60:40 to 55:45, depending on the nature of the chiral auxiliary (Scheme 2B). Surprisingly, no improvement in diastereoselectivity was observed by using the more hindered *gem*-disubstituted prolinol **8b**.

**Fig. 2:**
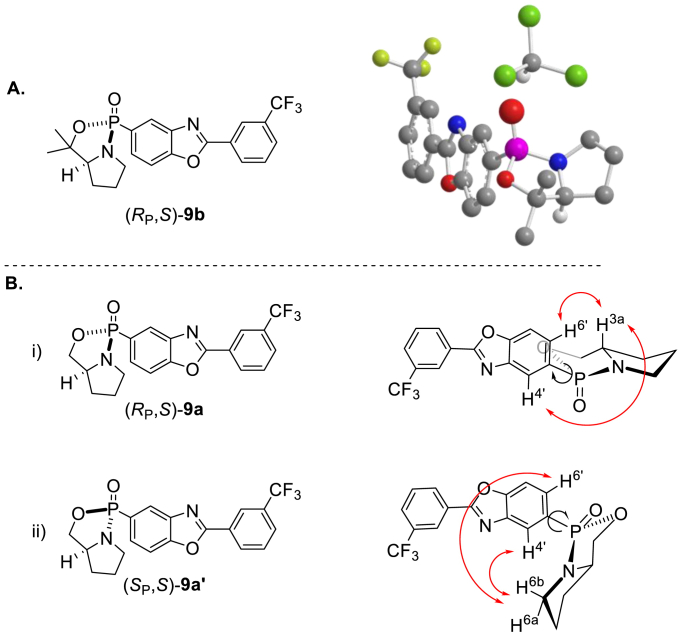
Determination of the relative configurations of (*R*_P_, *S*)-**9b** via single crystal X-ray diffraction analysis (2A) and (*R*_P_, *S*)-**9a** via NOESY analysis, observed NOE signals are depicted in red (2Bi); predicted NOE signals from diastereomer (*S*_P_, *S*)-**9a**′ which were not observed in the NOESY analysis (2Bii).

It is worth noting that both diastereoisomers ((*S*_P_, *S*)-**9a**′ and (*S*_P_, *S*)-**9b**′) spontaneously decomposed over a few days at ambient temperature leaving the diastereoisomers ((*R*_P_, *S*)-**9a** and (*R*_P_, *S*)-**9b**) as the only final products in 13% and 21% yields, respectively (See Scheme 2 and Fig. 2).

The relative configuration within the major diastereoisomer (*R*_P_, *S*)-**9b** was established unambiguously via single crystal X-ray diffraction analysis of the solvate (*R*_P_, *S*)-**9b**·CHCl_3_ [19]. The absolute (*R*_P_, *S*)-configuration of **9b** was assigned by reference to the known (*S*)-configuration of the prolinol auxiliary, and the determination of a Flack *x* parameter [20,21] of +0.013(10) for the structure of (*R*_P_, *S*)-**9b**·CHCl_3_ confirmed this assignment (Fig. 2A). The relative configuration within oxazaphospholidine (*R*_P_, *S*)-**9a** (Fig. 2B) was initially assigned by analogy to (*R*_P_, *S*)-**9b** and later supported by NOESY experiments (Supplementary Data). Given the rigid bicyclic structure of the oxazaphospholidine, it is plausible that protons H^4’^ and H^6’^ would display NOE signals with either H^3α^ or H^6β^, depending on the absolute configuration of the phosphorous atom. The NOESY spectrum exhibited NOE signals between H^4’^ and H^6’^ and H^3α^. No cross-peak with H^6β^ or any other hydrogens of the *Re* face of the prolinol ring was observed, consistent with the equatorial orientation of the benzoxazole ring (Fig. 2B).

**Scheme 3:**
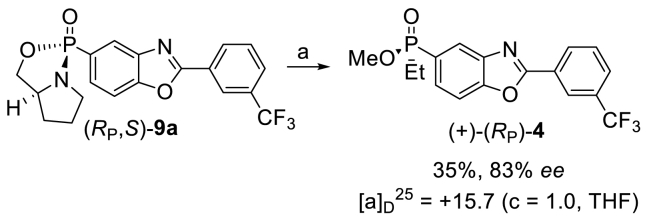
Synthesis of phosphinate (+)-(*R*_P_)-**4** from (*R*_P_, *S*)-**9a** by double inversion of configuration [14,18]. Reagents and conditions: (a) 1. EtMgBr, THF, rt, 16 h; 2. H_2_SO_4_ (0.1 M in MeOH), reflux, 1 h. (+)-(*R*_P_)-**4** [α]_D_^25^ = +15.7 (c = 1.0, THF), enantiomer 1 [α]_D_^25^ = +35.7 (c = 1.0, THF), enantiomer 2 [α]_D_^25^ = −38.3 (c = 1.0, THF) [Supplementary material].

With data to support the absolute and relative configurations of bicyclic oxaphospholidines **9a** and **9b**, ethylation of the two compounds was attempted (Scheme 3). Unfortunately, no alkylation of diastereoisomer (*R*_P_, *S*)-**9b** was achieved despite trialing a number of experimental conditions. When (*R*_P_, *S*)-**9a** was treated with EtMgBr in THF at rt for 16 h, the corresponding amidate was obtained in almost quantitative yield and in diastereomerically pure form.

The resulting phosphinamide of (*R*_P_, *S*)-**9a** was then subjected to acid-mediated methanolysis affording (+)-(*R*_P_)-**4** in 83% ee and 35% overall yield. The absolute configuration of (+)-(*R*_P_)-**4** was assigned by analogy with similar examples reported in the literature where a stereospecific overall inversion of configuration at phosphorus was unambiguously demonstrated [14,18]. The comparison of the HPLC traces and the comparable values for specific rotation supports enantiomer 1 as the enantiomer (+)-(*R*_P_)-**4** and enantiomer 2 as (−)-(*S*_P_)-**4**, enantiomer 1 and enantiomer 2 refers to the enantiomers’ relative retention times, Supplementary material.

### 2.2 In vitro and *in vivo* evaluation

Examination of the overall profile of SMT022357 and its enantiomers showed there are no significant differences between the biological activity of the two enantiomers and the properties were generally similar to those of the racemic mixture (Table 1). More specifically, the compound’s ability to increase utrophin’s transcription was assessed in the screening cell line H2K-*mdx* utrnA-luc, stably transfected with a 8.5 kb fragment of the human utrophin promoter linked to a firefly luciferase reporter gene [8,22]. The two enantiomers showed low micromolar activity in the reporter gene assay, which was comparable to that of the racemic mixture (Table 1). Similarly to the structurally related ezutromid [23], SMT022357 was found to inhibit firefly luciferase, though to a lesser extent. For this reason an orthogonal assay was used to verify the compounds’ activities. A homogenous time-resolved fluorescence (HTRF) assay was used to quantify human utrophin protein in cell lysates of iDMD cells, an immortalised muscle cell line derived from a DMD patient. The racemic mixture and the two enantiomers were found to increase the utrophin protein 1.2–1.3 fold (Supplementary data Fig. S1, Table S1).

**Table 1:** *In vitro* biological activity and ADME profiles of SMT022357 (±)-4 and enantiomers.

Cpd	H2K EC_50_±SEM^a^	Fluc IC_50_±SEM^b^	Sol^c^	logD^d^	Caco2		PPB f_u_ (%)^f^		Metabolic Stability (Hep)^g^			
					*P*_*app*_				m		h	
					A→B	ER^e^	m	h	CL_int_	T_1/2_	CL_int_	T_1/2_
(±)-**4**	1.28 ± 0.08	12.1 ± 0.1	468	3.80	18.0	0.91	2.97	2.77	ND^h^	ND	ND	ND
(+)-**4**	1.39 ± 0.06		295	4.02	ND	ND	2.35	4.16	16	89	<3	>460
(−)-**4**	1.20 ± 0.03		346	4.00	14.8	0.62	2.35	3.34	15	94	4.9	283

a RGA, reporter gene assay. EC_50_ determined in H2K-*mdx* utrnA-luc cells stably transfected with a 8.5 kb fragment of the human utrophin promoter linked to a firefly luciferase reporter gene (μM).

b Fluc, biochemical firefly luciferase inhibition assay (μM).

c Sol: Kinetic solubility (μM).

d logD measured with the shaking flask method.

e ER: extraction ratio.

f PPB: plasma protein binding reported as fraction unbound.

g Metabolic stability measured in hepatocytes and reported as intrinsic clearance CL_int_ (mL/min/10^6^ cells) and half-life T_1/2_ (min).

h ND, not determined.

In terms of physicochemical and ADME properties, the three compounds have good aqueous solubility and cell permeability, are not very lipophilic and show similar binding to plasma proteins. In addition, they are metabolically stable in human, but less so in mouse hepatocytes (Table 1).

Previous studies with the racemic mixture SMT022357 showed that oral administration of this compound led to increased expression of utrophin in skeletal and cardiac muscles, improving sarcolemmal stability and preventing the dystrophic pathology in *mdx* mice [8]. With the two enantiomers of SMT022357 in hand, we wanted to establish if there were any significant differences in their *in vivo* activity. The effect of the two enantiomers ((+)-**4**, (−)-**4**) was evaluated in 2 week old *mdx* mice, under the same protocol as before [8]. Briefly, the mice were dosed orally daily with 30 mg/kg ((+)-**4**, or (−)-**4** or vehicle) for 5 weeks. Immunofluorescence with a utrophin antibody revealed a higher utrophin protein signal after compound treatment and importantly that utrophin is localised at the sarcolemma in the extensor digitorum longus (EDL) muscle (Fig. S2).

**Fig. 3:**
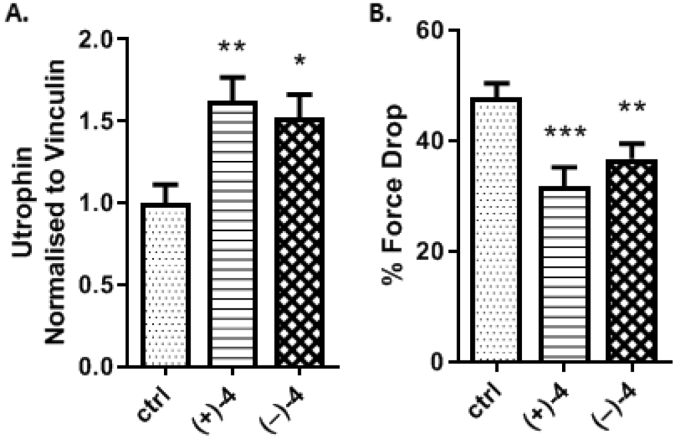
*In vivo* evaluation of SMT022357 enantiomers in *mdx* mice. A. The enantiomers of SMT022357 (±)**-4** increase utrophin expression in EDL muscles. 1.6-fold increase for (+)-**4**, and 1.5-fold increase with (−)-**4**. Values are mean ± SEM of n = 10 per group; *P < 0.05, **P < 0.01 B. EDL force drop (%). Reduction of force drop caused by eccentric contractions shows improved resistance of muscle to stress-induced damage. Values are mean ± SEM of n = 10 per group; **P < 0.01, ***P < 0.001.

Interestingly, the two enantiomers increased utrophin to a similar extent as demonstrated by quantification of the utrophin protein measured by western blot analysis. A 1.6-fold and 1.5-fold increase were observed for (+)-**4** and (−)-**4**, respectively (Fig. 3A). This is in good agreement with the results obtained with the racemic mixture in our previous study (1.8-fold increase of utrophin in EDL muscle) [8].

Next, we investigated functional improvements in the muscle upon administration of (+)-**4** and (−)-**4**. Dystrophin-deficient muscles are unable to sustain the mechanical stress induced by forced eccentric contractions and show an excessive force drop which is probably responsible for the activity-dependent damage seen in dystrophic muscles [24,25]. For this reason, the reduction of the force drop caused by eccentric contractions has been used as a quantitative test to measure the efficacy of therapeutic interventions, such as utrophin modulation. *Ex vivo* analysis of EDL muscles showed a substantial 34.8% (*p* = 0.00057) and 24.8% (*p* = 0.004) decrease in force drop compared with vehicle in (+)-**4** and (−)-**4** treated *mdx* mice, respectively (Fig. 3B). Taking into consideration that force is reduced only by 13.6% in wild type mice and up to 64.2% in *mdx* mice [26] (48.8% in the present experiment, Fig. 3) it is apparent that both enantiomers (+)-**4** and (−)-**4** significantly improved EDL muscle pathophysiology and ameliorated the DMD phenotype in *mdx* mice. These results, are consistent with the *in vivo* efficacy data previously reported for SMT022357 [8].

## 3 Conclusions

A second generation potential preclinical candidate for Duchenne muscular dystrophy with an improved physicochemical and ADME profile was presented. As the new analogue has a P-stereocentre, asymmetric synthesis was attempted to establish the absolute configurations of the two enantiomers. Further evaluation of the enantiomers *in vitro* and *in vivo* established that there are no significant differences in the biological and ADME profiles of the two. All in all, SMT022357 and enantiomers appear to have an improved ADME profile compared to ezutromid and improve the dystrophic phenotype in *mdx* mice.

## 4 Experimental section

### 4.1 General information

All NMR spectra were recorded on Bruker AV400 and AV500 spectrometers. ^1^H and ^13^C NMR spectra are reported as chemical shifts (*δ*) in parts per million (ppm) relative to the solvent peak using the Bruker internal referencing procedure (edlock). ^19^F NMR spectra are referenced relative to CFCl_3_ in CDCl_3_. Coupling constants (*J*) are reported in units of hertz (Hz). The following abbreviations are used to describe multiplicities – s (singlet), d (doublet), t (triplet), q (quartet), m (multiplet), br. (broad). High resolution mass spectra (HRMS, *m/z*) were recorded on a Bruker MicroTOF spectrometer using positive electrospray ionization (ESI^+^), on a Micromass GCT spectrometer using filed ionization (FI^+^) or chemical ionization (CI^+^), or on a Waters GCT Classic GCMS using electron impact ionization (EI). Infrared spectra were recorded either as the neat compound or in a solution using a Bruker Tensor 27 FT-IR spectrometer. Absorptions are reported in wavenumbers (cm^−1^). Melting points of solids were measured on an EZ-Melt apparatus and are uncorrected. Solvents were purchased from Sigma-Aldrich, Honeywell or Fisher. When dry solvents were required they were purified by expression through an activated alumina column built according to the procedures described by Pangborn and Grubbs. Chemicals were purchased from Acros, Alfa Aesar, Fisher, Fluorochem, Sigma-Aldrich and used as received. Reactions were monitored by thin-layer chromatography (TLC) carried out on Merck Kiesegel 60 F254 plates, silica gel column chromatography was performed over Merck silica gel C60 (40–60 μm).

### 4.2 Synthetic procedures

#### 4.2.1 5-Bromo-2-(3-(trifluoromethyl)phenyl)benzo[d]oxazole (2)

A mixture of 2-amino-4-bromophenol (384.3 g, 2.04 mol, 1.0 equiv.) and 3-(trifluoromethyl)benzoyl chloride (467 g, 2.25 mol, 1.1 equiv.) in toluene (3820 mL) was stirred at 55 °C for 3 h. The mixture was cooled to r.t. and CH_3_SO_3_H (78.6 g, 0.818 mol, 0.4 equiv.) was added. Then the mixture was stirred at reflux while the generated water was separated. After no more water was separated, the mixture was cooled at r.t. and partitioned between 5% NaCl (1900 mL) and EtOAc (1900 mL), extracted with EtOAc (2 x 950 mL), concentrated (to 1500 mL) and MeOH (3800 mL) was added partition. The generated precipitates were collected by filtration and then dried to give the desired product (506.9 g, 73%) as a pink solid. R_f_ = 0.80 (Pet. Ether:EtOAc = 10:1). mp: 127–128 °C. IR (ATR, neat): *ν*_max_ 2161, 1555, 1449, 1426, 1345, 1323, 1115, 1059, 910, 804, 694 cm^−1^. ^1^H NMR (400 MHz, CDCl_3_) *δ* 8.52 (s, 1H), 8.43 (d, *J* = 7.8 Hz, 1H), 7.94 (dd, *J* = 1.7, 0.7 Hz, 1H), 7.82 (d, *J* = 7.8 Hz, 1H), 7.69 (t, *J* = 7.8 Hz, 1H), 7.52 (dd, *J* = 8.6, 1.7 Hz, 1H), 7.50 (dd, *J* = 8.7, 0.8 Hz, 1H). ^13^C NMR (101 MHz, CDCl_3_) *δ* 162.6, 149.8, 143.5, 130.7 (q, *J* = 32.6 Hz), 130.8, 129.6, 128.7, 128.3 (q, *J* = 3.4 Hz), 127.6, 124.7 (q, *J* = 4.0 Hz), 123.5 (q, *J* = 273.0 Hz), 123.3, 117.7, 112.0. ^19^F NMR (376 MHz, CDCl_3_) *δ* −62.9. HRMS (ESI^+^) *m/z* calcd for C_14_H_8_BrF_3_NO [M+H]^+^: 341.97359 (^79^Br) - 343.97154 (^81^Br), found: 341.97378 (^79^Br) - 343.97154 (^81^Br).

#### 4.2.2 Ethyl(2-(3-(trifluoromethyl)phenyl)benzo[d]oxazol-5-yl)phosphinic acid (3)

The mixture of 5-bromo-2-(3-(trifluoromethyl)phenyl)-benzo[*d*]oxazole (450.11 g, 1.32 mol, 1.0 equiv.), EtP = (O)(OH) (148.64 g, 1.58 mmol, 1.2 equiv.) and DIPEA (255 g, 1.97 mol, 1.5 eq) in toluene (3.6 L) was purged with N_2_ for 10 min. And then xantphos (3.76 g, 6.45 mmol, 0.005 equiv.) and Pd(OAc)_2_ (1.49 g, 6.58 mmol, 0.005 equiv.) were added. The whole mixture was purged with N_2_ again and then heated to reflux for 21 h. The solvent was removed *in vacuo* and the residue was partitioned between EtOAc and NaOH (1 M). The aqueous layer was extracted with EtOAc again and then acidified with 1 M HCl and extracted with EtOAc. The combined organic layer was washed with brine and dried over Na_2_SO_4_ and concentrated *in vacuo*. The residue was purified by flash chromatography (CH_2_Cl_2_/MeOH = 20/1) to give the desired product (442.2 g, 94%) as a pink solid. R_f_ = 0.2 (DCM:MeOH = 10:1). ^1^H NMR (400 MHz, DMSO‑*d_6_*) *δ* 8.52 (d, *J* = 7.9 Hz, 1H), 8.46 (s, 1H), 8.15 (d, *J* = 11.5 Hz, 1H), 8.05 (d, *J* = 7.8 Hz, 1H), 7.97 (dd, *J* = 8.3, 1.9 Hz, 1H), 7.90 (t, *J* = 7.9 Hz, 1H), 7.86–7.78 (m, 1H), 1.89–1.77 (m, 2H), 0.96 (dt, *J* = 18.4, 7.6 Hz, 3H). ^13^C NMR (101 MHz, DMSO‑*d_6_*) *δ* 162.3, 152.6 (d, *J* = 3.1 Hz), 141.7 (d, *J* = 16.7 Hz), 131.8, 131.7 (d, *J* = 122.7 Hz), 131.3, 130.6 (q, *J* = 32.3 Hz), 129.3 (d, *J* = 11.1 Hz), 129.1 (q, *J* = 3.8 Hz), 127.6, 124.2 (q, *J* = 3.3 Hz), 124.1 (q, *J* = 273.0 Hz), 123.5 (d, *J* = 10.8 Hz), 111.9 (d, *J* = 13.5 Hz), 23.6 (d, *J* = 100.1 Hz), 6.7 (d, *J* = 4.8 Hz). ^31^P{^1^H} (162 MHz, DMSO‑*d_6_*) *δ* 37.9. ^19^F NMR (376 MHz, DMSO‑*d_6_*) *δ* −61.5.

#### 4.2.3 Methyl ethyl(2-(3-(trifluoromethyl)phenyl)benzo[d]oxazol-5-yl)phosphinate ((±)-4)

To a solution of ethyl(2-(3-(trifluoromethyl)phenyl)-benzo[*d*]oxazol-5-yl)phosphinic acid (400.7 g, 1.13 mol, 1.0 equiv.) in THF (4 L) and DMF (2.0 mL) COCl_2_ (167 g, 1.69 mol, 1.5 equiv.). The whole mixture was heated at 50 °C for 3 h. The reaction mixture was then cooled at r.t and aq 25% NaOMe (460 mL) was added while keeping the temperature at 10 °C. The whole mixture was allowed to warm to r.t. and stirred for 3 h. The layers were separated, the organic phase was washed with water, and concentrated *in vacuo.* Recrystallisation from EtOAc/heptanes gave the desired product (319.7 g, 95%) as a pink solid. R_f_ = 0.7 (silica gel, DCM: MeOH = 10 : 1, v/v). mp: 88–89 °C. IR (ATR, neat): *ν*_max_ 2947, 2160, 1627, 1557, 1459, 1427, 1343, 1324, 1242, 1174, 1138, 1041, 1020, 799, 688 cm^−1^. ^1^H NMR (400 MHz, DMSO‑*d_6_*) *δ* 8.49 (d, *J* = 7.9 Hz, 1H), 8.43 (s, 1H), 8.18 (ddd, *J* = 11.6, 1.4, 0.7 Hz, 1H), 8.04 (d, *J* = 7.9 Hz, 1H), 8.00 (ddd, *J* = 8.3, 2.4, 0.7 Hz, 1H), 7.88 (t, *J* = 7.9 Hz, 1H), 7.83 (ddd, *J* = 10.7, 8.3, 1.4 Hz, 1H), 3.54 (d, *J* = 10.9 Hz, 3H), 2.11–1.90 (m, 2H), 0.98 (dt, *J* = 19.0, 7.7 Hz, 3H). ^13^C NMR (101 MHz, DMSO‑*d_6_*) *δ* 162.1, 152.7 (d, *J* = 3.1 Hz), 141.5 (d, *J* = 16.8 Hz), 131.4, 130.9, 130.1 (q, *J* = 32.5 Hz), 129.3 (d, *J* = 11.1 Hz), 128.7(q, *J* = 3.1 Hz), 127.2 (d, *J* = 120.1 Hz), 127.1, 123.72 (d, *J* = 4.0 Hz), 123.71 (d, *J* = 11.3 Hz), 123.67 (q, *J* = 272.7 Hz), 112.9 (d, *J* = 13.6 Hz), 50.8, 21.2 (d, *J* = 101.6 Hz), 5.8 (d, *J* = 4.7 Hz). ^31^P{^1^H} (162 MHz, DMSO‑*d_6_*) *δ* 46.7. ^19^F (376 MHz, DMSO‑*d_6_*) *δ* −61.6. HRMS (ESI^+^) *m/z* calcd for C_17_H_16_F_3_NO_3_P [M+H]^+^: 370.08144, found: 370.08084.

#### 4.2.4 Diethyl (2-(3-(trifluoromethyl)phenyl)benzo[d]oxazol-5-yl)phosphonate (6)

The mixture of 5-bromo-2-(3-(trifluoromethyl)phenyl)-benzo[*d*]oxazole (2.0 g, 5.84 mmol, 1.0 equiv.), HP(O)(OEt)_2_ (830 μL, 6.43 mmol, 1.1 equiv.) and NEt_3_ (900 μL, 6.43 mmol, 1.1 equiv.) in toluene (20 mL) was purged with N_2_ for 10 min. Pd(PPh_3_)_4_ (337 mg, 0.292 mmol, 5.0 mol%) was added, the whole mixture was purged with N_2_ again and then heated to reflux for 3 h Et_2_O was then added to the reaction mixture and the insoluble solid was filtered. The solid was washed with Et_2_O (x 2) and dried under vacuum. The residue was purified by flash chromatography (Pentane/Acetone = 9/1) to give the desired product as a brown solid in quantitative yield. mp: 223-125 °C. IR (ATR, neat): *ν*_max_ 2980, 2160, 1629, 1420, 1346, 1326, 1247, 1164, 1128, 1048, 1019, 963, 948, 796, 691 cm^−1^. ^1^H NMR (400 MHz, CDCl_3_) *δ* 8.54 (s, 1H), 8.46 (d, *J* = 7.8 Hz, 1H), 8.26 (ddd, *J* = 13.8, 1.4, 0.7 Hz, 1H), 7.90 (ddd, *J* = 12.8, 8.4, 1.4 Hz, 1H), 7.83 (d, *J* = 7.7 Hz, 1H), 7.74–7.66 (m, 2H), 4.27–4.06 (m, 4H), 1.35 (td, *J* = 7.1, 0.6 Hz, 6H). ^13^C NMR (126 MHz, CDCl_3_) *δ* 162.6, 153.2 (d, *J* = 3.3 Hz), 142.0 (d, *J* = 20.7 Hz), 131.7 (q, *J* = 32.9 Hz), 130.8, 129.7, 129.4 (d, *J* = 11.9 Hz), 128.4 (q, *J* = 3.9 Hz), 127.4, 126.4, 124.7 (q, *J* = 4.0 Hz), 124.5 (d, *J* = 10.8 Hz), 123.5 (q, *J* = 272.7 Hz), 111.2 (d, *J* = 17.0 Hz), 62.3 (d, *J* = 5.5 Hz), 16.3 (d, *J* = 6.4 Hz). ^31^P{^1^H} (162 MHz, CDCl_3_) *δ* 18.2. HRMS (ESI^+^) *m/z* calcd for C_18_H_18_F_3_NO_4_P [M+H]^+^: 400.09201, found: 400.09180.

#### 4.2.5 (2-(3-(Trifluoromethyl)phenyl)benzo[d]oxazol-5-yl)phosphonic dichloride (7)

To a solution of diethyl (2-(3-(trifluoromethyl)phenyl)-benzo[*d*]oxazol-5-yl)phosphonate (500 mg, 1.25 mmol) in SOCl_2_ (600 μL) was added 2 drops of DMF. The whole mixture was heated to 90 °C overnight. The SOCl_2_ was removed *in vacuo* affording (2-(3-(trifluoromethyl)phenyl)benzo[*d*]oxazol-5-yl)phosphonic dichloride as a yellow oil in quantitative yield. The crude oil was used in the next step without any further purification. ^1^H NMR (500 MHz, CDCl_3_) *δ* 8.49 (d, *J* = 7.7 Hz, 1H), 8.44 (dd, *J* = 18.5, 1.5 Hz, 1H), 8.05 (ddd, *J* = 16.9, 8.5, 1.7 Hz, 1H), 7.87 (d, *J* = 7.8 Hz, 1H), 7.83 (dd, *J* = 8.5, 4.9 Hz, 1H), 7.73 (t, *J* = 7.8 Hz, 2H). ^13^C NMR (126 MHz, CDCl_3_) *δ* 163.8, 154.2 (d, *J* = 4.3 Hz), 142.2 (d, *J* = 26.8 Hz), 131.9 (q, *J* = 33.1 Hz), 131.2 (d, *J* = 159.4 Hz), 131.1, 129.8, 129.0 (q, *J* = 3.8 Hz), 127.9 (d, *J* = 16.3 Hz), 126.9, 124.9 (q, *J* = 3.9 Hz), 123.5 (d, *J* = 16.5 Hz), 123.5 (q, *J* = 272.8 Hz), 111.9 (d, *J* = 21.6 Hz). ^31^P{^1^H} (162 MHz, CDCl_3_) *δ* 35.0.

#### 4.2.6 (1R,3aS)-1-(2-(3-(Trifluoromethyl)phenyl)benzo[d]oxazol-5-yl)tetrahydro-3H-pyrrolo[1,2-c][1,3,2]oxazaphosphole 1-oxide (R_P_**,**S)-9a

The mixture of (2-(3-(trifluoromethyl)-phenyl)benzo[*d*]oxazol-5-yl)phosphonic dichloride (2.15 g, 5.66 mmol, 1.0 equiv.) and (*S*)-prolinol (560 μL, 5.66 mmol, 1.0 equiv.) in THF (25 mL) was added NEt_3_ (1.6 mL, 11.32 mmol, 5.0 equiv.) at 0 °C. The whole mixture was stirred at rt until TLC showed the reaction was completed. Et_2_O was then added to the reaction mixture and the insoluble solid was filtered. The solid was washed with Et_2_O (x 2) and dried under vacuum. The corresponding 1-(2-(3-(trifluoromethyl)phenyl)-benzo[*d*]oxazol-5-yl)tetrahydro-3*H*-pyrrolo[1,2-*c*][1,3,2]oxazaphosphole 1-oxide was obtained as a 60 : 40 mixture of **(*R***_**P**_**, *S*)-9a** and **(*S***_**P**_**, *S*)-9a**. The isomer (*R*_P_, *S*)-**9a** (301 mg, 13%) was isolated by flash chromatography (Pentane/EtOAc/NEt_3_ = 14/85/1) as a white solid. mp: 227–229 °C. ^1^H NMR (500 MHz, CDCl_3_) *δ* **(*R***_P_, ***S*)-9a:** 8.54 (s, 1H)H^13’^, 8.46 (d, *J* = 7.9 Hz, 1H) H^9’^, 8.29–8.23 (m, 1H) H^4’^, 7.93 (ddd, *J* = 13.2, 8.4, 1.5 Hz, 1H) H^6’^, 7.83 (d, *J* = 7.7 Hz, 1H) H^11’^, 7.75–7.64 (m, 2H) H^7’^+ H^10’^, 4.39 (ddd, *J* = 20.3, 8.8, 6.6 Hz, 1H) H^3α^, 4.20 (dqd, *J* = 8.6, 6.9, 4.4 Hz, 1H) H^6a^, 3.96 (td, *J* = 8.7, 2.4 Hz, 1H) H^3β^, 3.85–3.73 (m, 1H) H^6α^, 2.97 (ddt, *J* = 14.5, 10.4, 7.4 Hz, 1H) H^6β^, 2.14–1.98 (m, 3H) H^5α^ + H^5β^ + H^4α^, 1.88–1.80 (m, 1H) H^4β^; **(*S***_P_, ***S*)-9a’:** 8.55 (s, 1H), 8.47 (d, *J* = 7.6 Hz, 1H), 8.12 (d, *J* = 13.6 Hz, 1H), 7.96–7.89 (m, 1H), 7.84 (d, *J* = 8.4 Hz, 1H), 7.78–7.71 (m, 2H), 4.79 (ddd, *J* = 15.0, 9.1, 6.4 Hz, 1H), 4.32 (m, 1H), 4.12 (td, *J* = 8.6, 4.1 Hz, 1H), 3.12–3.05 (m, 1H), 2.94–2.87 (m, 1H), 2.14–1.79 (m, 3H), 1.70–1.60 (m, 1H)·^13^C NMR (126 MHz, CDCl_3_) *δ* **(*R***_P_, ***S*)-9a:** 162.6 (C^2^’), 153.1 (d, *J* = 3.4 Hz) (C^7a^’), 141.9 (d, *J* = 21.1 Hz) (C^3a^’), 131.7 (q, *J* = 33.0 Hz) (C^12^’), 130.8 (C^10^’), 129.7 (C^9^’), 129.4 (d, *J* = 12.3 Hz) (C^6^’), 128.4 (q, *J* = 3.7 Hz) (C^13^’), 127.9 (d, *J* = 185.1 Hz) (C^5^’), 127.5 (C^8^’), 124.7 (q, *J* = 3.9 Hz) (C^11^’), 124.3 (d, *J* = 11.7 Hz) (C^4^’), 123.7 (q, *J* = 272.6 Hz) (C^14^’), 111.0 (d, *J* = 17.5 Hz) (C^7^’), 69.8 (d, *J* = 2.3 Hz) (C^3^), 63.3 (d, *J* = 7.6 Hz) (C^3a^), 45.4 (C^6^), 30.0 (d, *J* = 3.3 Hz) (C^5^), 27.6 (d, *J* = 1.9 Hz) (C^4^). ^31^P{^1^H} (162 MHz, CDCl_3_) *δ* **(*R***_**P**_**, *S*)-9a:** 38.3; **(*S***_**P**_**, *S*)-9a’:** 32.9.

#### 4.2.7 (1R,3aS)-3,3-Dimethyl-1-(2-(3-(trifluoromethyl)phenyl)benzo[d]oxazol-5-yl)tetrahydro-3H-pyrrolo[1,2-c][1,3,2]oxazaphosphole 1-oxide ((R_P_**,**S)-9b)

The mixture of (2-(3-(trifluoromethyl)phenyl)-benzo[*d*]oxazol-5-yl)phosphonic dichloride (250 mg, 1.25 mmol, 1.0 equiv.) and (*S*)-2-(pyrrolidin-2-yl)propan-2-ol hydrochloride (207 mg, 1.25 mmol, 1.0 equiv.) in THF (5 mL) was added NEt_3_ (696 μL, 5.0 mmol, 4.0 equiv.) at 0 °C. The whole mixture was stirred at rt until TLC showed the reaction was completed. Et_2_O was then added to the reaction mixture and the insoluble solid was filtered. The solid was washed with Et_2_O (x 2) dried under vacuum. The corresponding 3,3-dimethyl-1-(2-(3-(trifluoromethyl)phenyl)-benzo[*d*]oxazol-5-yl)tetrahydro-3*H*-pyrrolo[1,2*c*][1,3,2]oxaza-phosphole 1-oxide was obtained as a 57 : 43 mixture of **(*R***_**P,**_ ***S*)-9b** and **(*S***_**P,**_ ***S*)-9b**′. The two isomers were isolated by flash chromatography (Pentane/EtOAc = 15/85) to give **(*R***_**P,**_ ***S*)-9b** (113 mg, 21%) and **(*S***_**P,**_ ***S*)-9b**′ (112 mg, 21%) both as white solids. Crystallisation of **(*R***_**P,**_ ***S*)-9b** from MeOH. ^1^H NMR (400 MHz, CDCl_3_) *δ* 8.47 (s, 1H), 8.38 (d, *J* = 7.9 Hz, 1H), 8.23–8.11 (m, 1H), 7.86 (ddd, *J* = 13.2, 8.3, 1.5 Hz, 1H), 7.75 (d, *J* = 7.8 Hz, 1H), 7.67–7.55 (m, 3H), 3.88 (td, *J* = 7.0, 5.4 Hz, 1H), 3.70–3.60 (m, 1H), 2.99–2.87 (m, 1H), 2.13–1.99 (m, 1H), 1.97–1.85 (m, 2H), 1.86–1.74 (m, 1H), 1.46 (s, 3H), 1.42 (s, 3H). ^13^C NMR (126 MHz, CDCl_3_) *δ* 161.5, 151.9 (d, *J* = 3.4 Hz), 140.8 (d, *J* = 21.0 Hz), 131.1, 130.7 (q, *J* = 33.0 Hz), 129.8, 128.7 (d, *J* = 1.9 Hz), 127.8 (d, *J* = 184.9 Hz), 127.5 (d, *J* = 12.2 Hz), 127.3 (q, *J* = 3.7 Hz), 123.6 (q, *J* = 3.7 Hz), 123.1 (d, *J* = 11.6 Hz), 122.6 (q, *J* = 272.4 Hz), 109.9 (d, *J* = 17.3 Hz), 83.0, 73.0 (d, *J* = 8.0 Hz), 44.4 (d, *J* = 2.2 Hz), 28.3 (d, *J* = 3.5 Hz), 27.7 (d, *J* = 4.1 Hz), 24.0. ^31^P{^1^H} (162 MHz, CDCl_3_) *δ* 31.9.

#### 4.2.8 (1S,3aS)-3,3-Dimethyl-1-(2-(3-(trifluoromethyl)phenyl)benzo[d]oxazol-5-yl)tetrahydro-3H-pyrrolo[1,2-c][1,3,2]oxazaphosphole 1-oxide ((S_P_**,**S)-9b′)

^1^H NMR (400 MHz, CDCl_3_) *δ* 8.54 (s, 1H), 8.45 (d, *J* = 7.9 Hz, 1H), 8.15 (d, *J* = 13.0 Hz, 1H), 7.95 (ddd, *J* = 12.3, 8.3, 1.5 Hz, 1H), 7.83 (d, *J* = 8.1 Hz, 1H), 7.76–7.63 (m, 2H), 3.87 (td, *J* = 9.7, 5.7 Hz, 1H), 3.16 (dq, *J* = 10.4, 7.5 Hz, 1H), 3.02 (dq, *J* = 10.4, 7.5 Hz, 1H), 1.88 (m, 3H), 1.74 (s, 3H), 1.70–1.57 (m, 1H), 1.52 (s, 3H). ^13^C NMR (126 MHz, CDCl_3_) *δ* 162.7, 153.1 (d, *J* = 3.3 Hz), 142.1 (d, *J* = 20.1 Hz), 131.8 (q, *J* = 33.2 Hz), 130.9 (d, *J* = 11.7 Hz), 130.8, 129.7, 128.5 (q, *J* = 3.8 Hz), 127.4, 125.6 (d, *J* = 167.1 Hz), 124.7 (q, *J* = 4.0 Hz), 124.4 (d, *J* = 11.6 Hz), 123.6 (q, *J* = 272.7 Hz), 111.2 (d, *J* = 16.3 Hz), 85.5 (d, *J* = 2.9 Hz), 72.3 (d, *J* = 10.8 Hz), 43.6 (d, *J* = 4.6 Hz), 30.5 (d, *J* = 2.0 Hz), 27.5 (d, *J* = 4.4 Hz), 27.1 (d, *J* = 4.6 Hz), 24.4 (d, *J* = 4.8 Hz). ^31^P{^1^H} (162 MHz, CDCl_3_) *δ* 29.3.

#### 4.2.9 Synthesis of methyl (R)-ethyl(2-(3-(trifluoromethyl)phenyl)benzo[d]oxazol-5-yl)phosphinate by double inversion of configuration. ((R_P_)-(+)-4)

EtMgBr (1.0 M in THF) (1.22 mL, 1.22 mmol, 5.0 equiv.) was added dropwise at 0 °C to a solution of (1*R*,3a*S*)-1-(2-(3-(trifluoromethyl)phenyl)benzo[*d*]oxazol-5-yl)tetrahydro-3*H*-pyrrolo[1,2-*c*][1,3,2]oxazaphosphole 1-oxide (95.0 mg, 0.23 mmol, 1.0 equiv.) in THF (1.0 mL). The whole mixture was stirred at rt until TLC showed the reaction was completed. The solution was then quenched with aq NH_4_Cl and extracted with EtOAc (3 × 2 mL), and the combined organic layers were washed with brine, dried over MgSO_4_, and concentrated *in vacuo*.

The resulting crude mixture was dissolved in a 0.1 M solution of H_2_SO_4_ in MeOH (1.0 mL) and heated at 75 °C for 1 h. Then the suspension was quenched with a saturated aqueous solution of NaHCO_3_ and extracted with EtOAc (3 × 2 mL). The combined organic layers were washed with brine, dried over MgSO_4_, and concentrated *in vacuo*. Methyl (*R*)-ethyl(2-(3-(trifluoromethyl)phenyl)-benzo[*d*]oxazol-5-yl)phosphinate was isolated by column chromatography (2% MeOH in DCM) as a white solid (18.0 mg, 35% over two steps).

### 4.3 In vitro assays

#### 4.3.1 H2K reporter gene assay

##### 4.3.1.1 Cell culture

H2K-*mdx* utrnA-luc cells [27] were maintained in DMEM (Invitrogen) supplemented with 20% Fetal Bovine Serum Gold (PAA), 5% CEE (SLI), 2 mM l-Glutamine (Invitrogen), 1% Penicillin Streptomycin (Invitrogen) and 2 μg/500 mL Mouse Interferon-γ (Roche). Cells were maintained at 10% CO_2_ at 33 °C. EC_50_ is the average from three or more biological replicates, 3 technical replicates each.

##### 4.3.1.2 Utrophin firefly luciferase reporter gene assay

White flat bottomed 96 well plates (Corning) were seeded with 10000 H2K-*mdx* utrnA-luc cells. After 24 h at 10% CO_2_ and 33 °C, the cells were dosed with compound in triplicate from 10 mM solution stocks in DMSO (final DMSO concentration was 0.3%). Compounds were diluted in the following concentration series: 0 μM, 0.01 μM, 0.1 μM, 0.3 μM, 1 μM, 3 μM, 10 μM, 30 μM. The cells were incubated for a further 24 h, (10% CO_2_, 33 °C). Relative luminescence readout after using the Luciferase Assay System (Promega, E1501) reagents was measured using a FLUOstar Optima plate reader (BMG Labtech). The means from the biological triplicates were fitted with a four parameter logistic function with least squares regression (Levenberg-Marquardt algorithm) to calculate EC_50_ values.

##### 4.3.1.3 Firefly luciferase biochemical inhibition assay

Recombinant firefly luciferase (QuantiLum Promega, E1701) was assayed at a final concentration of 0.6 nM in a buffer containing 25 mM HEPES, 5 mM MgCl_2_, 1 mM EDTA, 5 mM DTT and 1 mg/mL BSA. Compounds were diluted in the following concentration series: 0 nM, 0.95 nM, 3 nM, 30 nM, 95 nM, 0.3 μM, 0.95 μM, 3 μM, 9.5 μM, 30 μM, from 10 mM stocks in DMSO (with a final assay concentration of DMSO at 0.3%). PTC124 was used as a positive control [28]. Luciferase substrates ATP (Sigma) and D-luciferin (Promega) were used in a final assay concentration of 10 μM, close to their K_M_ concentrations [29]. Luciferase was pre-incubated with ATP and the query compound at 0 °C for 15 min. D-Luciferin was dispensed and endpoint luminescence output immediately read using a FLUOstar Optima plate reader (BMG Labtech). Luminescence output was fitted with a four parameter logistic function with least squares regression (Levenberg-Marquardt algorithm) to calculate IC_50_ values. IC_50_ is the average of two biological replicates, three technical replicates each.

#### 4.3.2 HTRF assay: utrophin quantification (Evotec)

The human utrophin HTRF kit was obtained from Cisbio Bioassay (Parc Marcel Boiteux, 30200 Codolet, France). In this, utrophin is detected in a sandwich assay format using two different specific antibodies, one labelled with Eu^3+^-Cryptate (donor) and the second with d2 (acceptor). When the donor/acceptor pair is in close proximity, the excitation of the donor with a light source (laser or flash lamp) triggers a Fluorescence Resonance Energy Transfer (FRET) towards the acceptor, which in turn fluoresces at a specific wavelength (665 nm). The measurement of HTRF emissions at two different wavelengths (620 nm for the donor and 665 nm for the acceptor) allows the ratiometric reduction of data to correct for well-to-well variability and signal quenching from assay components and media.

The specific signal modulates positively in proportion to human utrophin. The assay is run under a two-plate assay protocol as follows: cells are plated, stimulated and lysed in a first culture plate, then lysates are transferred to the assay plate for utrophin detection using HTRF reagents. This protocol gives the ability to monitor the cell viability and confluence.

##### 4.3.2.1 Cell culture

Immortalised DMD myoblasts isolated from the Fascia lata muscle of a 10 year old male, del 52 DMD (KM571DMD10FL) were acquired through collaboration with Professor Vincent Mouly (Insitut de Myologie, Paris). These were cultured in Skeletal Muscle Cell Growth Medium and Supplement (PromoCell C-23060), 20% Fetal Bovine Serum (Life Technologies) and 1% Penicillin Streptomycin (Life Technologies). Cells were maintained at 5% CO_2_ at 37 °C.

###### 4.3.2.1.1 Growth cell culture conditions

Cells were maintained in Ham’s F10 nutrient mix supplemented with 20% FCS, dexamethasone (1 μM), Rh fibroblast growth factor (10 ng/mL), and penicillin/streptomycin (100 U/mL). The cells were grown in 50 mL growth medium/T225 cm^2^ flask (Corning).

###### 4.3.2.1.2 Starvation conditions

DMEM F12, KnockOut Serum Replacement (5%), dexamethasone (1 μM), antibiotics/antimycotics (1X), HEPES (20 mM).

###### 4.3.2.1.3 Subculturing

The medium was removed and the cell monolayer was rinsed twice with DPBS (w/o Ca/Mg). Remove DPBS and add 3 mL of Accutase. Incubate at 37 °C until the cells detach (approximately 2–3 min). Add 17 mL of fresh growth medium to homogenize the suspension. Cells are centrifuged (5 min, 1200 rpm) and suspended in 15 mL of fresh growth medium. Add the proper volume of cell suspension (1/3 to 1/10) to a new T225 cm^2^ flask. Cells were maintained at 5% CO_2_ at 37 °C, 90% RH.

The cells were plated in 384-well plates (50 μL/well, 8000 cells/well, 5 replicates for each condition evaluated). After 24 h at 37 °C, 5% CO_2_, 90% RH the cells were serum starved to get synchronised and the next day the cells were dosed with compounds in triplicate from stock solutions (medium, 0.3% DMSO, cpd 10 μM) and incubated for 120 h. The cells were then lysed, a mix of the two antibodies (Eu^3+^/d_2_) was added and incubated for further 24 h. Time-resolved fluorescence was measured on a EnVision™ 2103 Multilabel plate reader set up for Eu^3+^-cryptate and fluorescence emission was measured at 665 nm and 620 nm.

Results are expressed as HTRF ratio (mean ± standard deviation). Analysis of the raw data was performed using GraphPad Prism 7 software, and data was then compared to the untreated cells. Calculations were performed on Excel software. Statistical analysis was performed with GraphPad Prism 7 software using the unpaired *t*-test (2 biological replicates, 3 technical replicates each).

### 4.4 In vivo assays

#### 4.4.1 Mice and drug treatment

15 day-old male mdx (C57BL/10ScSn-Dmdmdx/J; Charles River Laboratories, MA, USA) littermates were randomly assigned and treated with (±)-**4**, (+)-**4**, (−)-**4** (30 mg/kg) or vehicle only [phosphate buffered saline (PBS), 0.1% Tween-20, 5% DMSO] by daily intraperitoneal injection for 1 week and then daily oral gavage for a further 4 weeks. At the end of drug treatment, mice were sacrificed by CO_2_ asphyxiation in accordance with Schedule I of the UK Animals (Scientific Procedures) Act 1986 and muscle and blood samples taken.

#### 4.4.2 Histology

Frozen TA muscle sections (10 μm thick) were air dried for 10 min before being placed in hematoxylin solution (Sigma, UK #GHS232) for 8 min, slides were washed in tap water to remove excess stain and placed in 70% ethanol, 1% HCl for 10 s followed by 5 s in 1% Eosin Y solution (Sigma, UK #E4382) in 80% ethanol. Following a series of alcohol washes unbound stain was removed in Histochoice clearing agent (Sigma UK #H2779) and mounted using Histomount (National Diagnostics, UK #HS-103). Slides were examined under an Axioplan 2 Microscope System (Carl Zeiss, Germany) to obtain images.

#### 4.4.3 Immunofluorescence histology

Frozen TA muscle sections (10 μm thick) were fixed in acetone for 10 min, washed for 5 min in PBS and blocked for 1 h in MOM blocking solution (M.O.M.™ Kit, FMK-2202. Vector Laboratories). Following 2 × 2 min washes, sections were incubated overnight with primary antibodies at 4 °C. The following antibody and dilutions were used: goat polyclonal anti-utrophin (1:500, URD40). After 2 × 2 min washes and 5 min in MOM diluent (M.O.M.™ Kit, FMK-2202. Vector Laboratories), sections were incubated in anti-goat (1:2000; A11055, Life Technologies) Alexa Fluor® 488 secondary antibody for 2 h at room temperature. Sections were examined under an Axioplan 2 Microscope System (Carl Zeiss, Germany) and multi-acquisition module used to obtain images.

#### 4.4.4 Isolated muscle function analysis

Peak force, specific force and force drop from the EDL muscle were measured as previously described [8]. In brief, isolated EDL were attached to a lever arm connected to a force transducer and stimulator; the equipment was controlled using the signal interface and the DMC software (Aurora Scientific, Bristol, United Kingdom). After determination of the optimum length (*L*_o_), the optimum fibre length (*L*_f_) was calculated by multiplying *L*_o_ by the fibre length to muscle length ratio of 0.44. A force–frequency curve was generated and the maximum isometric force calculated. Absolute force (*P*_o_) are normalized to specific force (s*P*_o_; mN/cm^2^) using the equation (muscle mass/L_f_ ×1.06). Percentage force drop was calculated by comparing maximum force between the first (EC0) and fifth (EC5), expressed as a percentage of EC0. All data were digitized and analysed using the DMC software.

#### 4.4.5 Protein analyses

Muscles samples were homogenized (Precellys 24 Bertin Technologies) for 2 × 30 s 5500 rpm in chilled RIPA buffer (R0278-50 mL, Sigma-Aldrich) supplemented with protease inhibitors (1:100; P8340, Sigma-Aldrich). Following BCA quantification (23227; ThermoFisher Scientific), 30 μg of total protein were heat-denatured for 5 min at 100 °C before loading onto NuPAGE 3–8% TRIS Acetate Midi Gel (Novex, Life Technologies) and transferred to PVDF membranes (Millipore). Membranes were blocked for 1 h with Odyssey Blocking buffer (926–41090; LI-COR; USA) and then incubated with primary antibodies in Odyssey Blocking buffer PBS + 0.1% Tween for 2 h at room temperature. Primary antibodies used were: mouse monoclonal anti-utrophin (1:50, MANCHO3(84A), gift from G.E. Morris), mouse monoclonal anti-MYH3 (1:100, sc-53091, Santa Cruz Biotechnology, USA), anti-dystrophin (1:200, ab15277, Abcam UK), α-actinin (1:400, sc-7453, Santa Cruz Biotechnology, USA), anti-GAPDH (1:20000, #MA5-15738, Invitrogen UK) The Odyssey Imaging System (LI-COR Biosciences; USA) was used to read infrared fluorescence of the secondary antibodies. Protein was quantified using Image Studio Lite Ver 5.0 software (LI-COR Biosciences; USA).

## Notes

D.E., S.H., N.R., J.M.T., F.X.W. were Summit Therapeutics plc employees or consultants thereof at the time the work was conducted.
