# Supplementary material for: Synthesis of SMT022357 enantiomers and in vivo evaluation in a Duchenne muscular dystrophy mouse model
Source: Tetrahedron. 2020 Jan 10;76(2):130819. doi: 10.1016/j.tet.2019.130819 (PMC7369641; doi:10.1016/j.tet.2019.130819)

**Synthesis of SMT022357 enantiomers and *in vivo* evaluation in mdx mice**

Arran Babbs,^a^ Adam Berg,^a^ Maria Chatzopoulou,^b^ Kay E. Davies,^a^ Stephen G. Davies,^b^ Benjamin Edwards,^a^ David J. Elsey,^c^ Enrico Emer,^b^ Aude L. A. Figuccia,^b^ Ai M. Fletcher,^b^ Simon Guiraud,^a^ Shawn Harriman,^c^ Lee Moir,^a^ Neil Robinson,^d^ Jessica A. Rowley,^b^ Angela J. Russell,^b,e^ Sarah E. Squire,^a^ James E. Thomson,^b^ Jonathon M. Tinsley,^c^ Francis X. Wilson,^c^ Graham M. Wynne^b^

^a^ Department of Anatomy and Genetics, MDUK Oxford Neuromuscular Centre, University of Oxford, Oxford OX1 3PT, UK

^b^ Department of Chemistry, University of Oxford, Chemistry Research Laboratory, Mansfield Road, Oxford, OX1 3TA, UK

^c^ Summit Therapeutics plc, 136a Eastern Avenue, Milton Park, Abingdon, OX14 4SB, UK

^d^ S.H.B. Enterprises Ltd, 55 Station Road, Beaconsfield, HP19 1QL

^e^ Department of Pharmacology, University of Oxford, Mansfield Road, Oxford, OX1 3PQ, UK

**Separation of the two enantiomers by prep-HPLC**

Racemic (±)-**4** (SMT022357, 1010 mg) was dissolved to 100 mg/mL in EtOH and was then purified by HPLC. Combined fractions of each of (+)-**4** and (–)-**4** were then evaporated to near dryness using a rotary evaporator, transferred into final vessels with DCM, which was removed under a stream of nitrogen at 40C before being stored in a vacuum oven at 40 ^o^C and 5 mbar for 16 h to afford (+)-**4** (473 mg, 47% yield) and (–)-**4** (451 mg, 45% yield) as light brown powders.

Purification conditions:

| Column: | Lux Cellulose-4 (21.2 mm x 250 mm, 5 μm) |
| --- | --- |
| Column temperature: | ambient |
| Flow rate: | 21 mL/min |
| Detector Wavelength: | 220 nm |
| Injection Volume: | 1000 μL 100 mg |
| Isocratic conditions | 50:50 heptane/EtOH |

Chiral purity analysis conditions:

| Column: | Lux Cellulose-4 (4.6 mm x 250 mm, 5 μm) |
| --- | --- |
| Column temperature: | ambient |
| Flow rate: | 1 mL/min |
| Detector Wavelength: | 254 nm |
| Injection Volume: | 1.0 μL |
| Isocratic conditions | 50:50 heptane/EtOH |

Chemical purity analysis conditions:

| Column Details | XSelect CSH C18 (50 x 2.1 mm, 1.7 μm) | | |
| --- | --- | --- | --- |
| Column Temperature | 40 ^o^C | | |
| Flow Rate | 0.6 mL/min | | |
| Detector Wavelength | 240 nm | | |
| Injection Volume | 1.0 μL | | |
| Mobile Phase A | 0.1% TFA (aq) | | |
| Mobile Phase B | 0.1% TFA in acetonitrile | | |
| Gradient Profile | Time (mins) | %A | %B |
|  | 0 | 95 | 5 |
|  | 4 | 5 | 95 |
|  | 4.02 | 0 | 100 |
|  | 4.5 | 0 | 100 |
|  | 4.52 | 95 | 5 |
|  | 6 | 95 | 5 |

PDA chromatogram for (±)-**4.**

PDA Peak Results:

|  | Retention Time (min) | Area (μV×sec) | % Area | Width (sec) |
| --- | --- | --- | --- | --- |
| 1 | 4.798 | 4.894e3 | 49.97 | 0.130 |
| 2 | 6.239 | 4.901e3 | 50.03 | 0.186 |


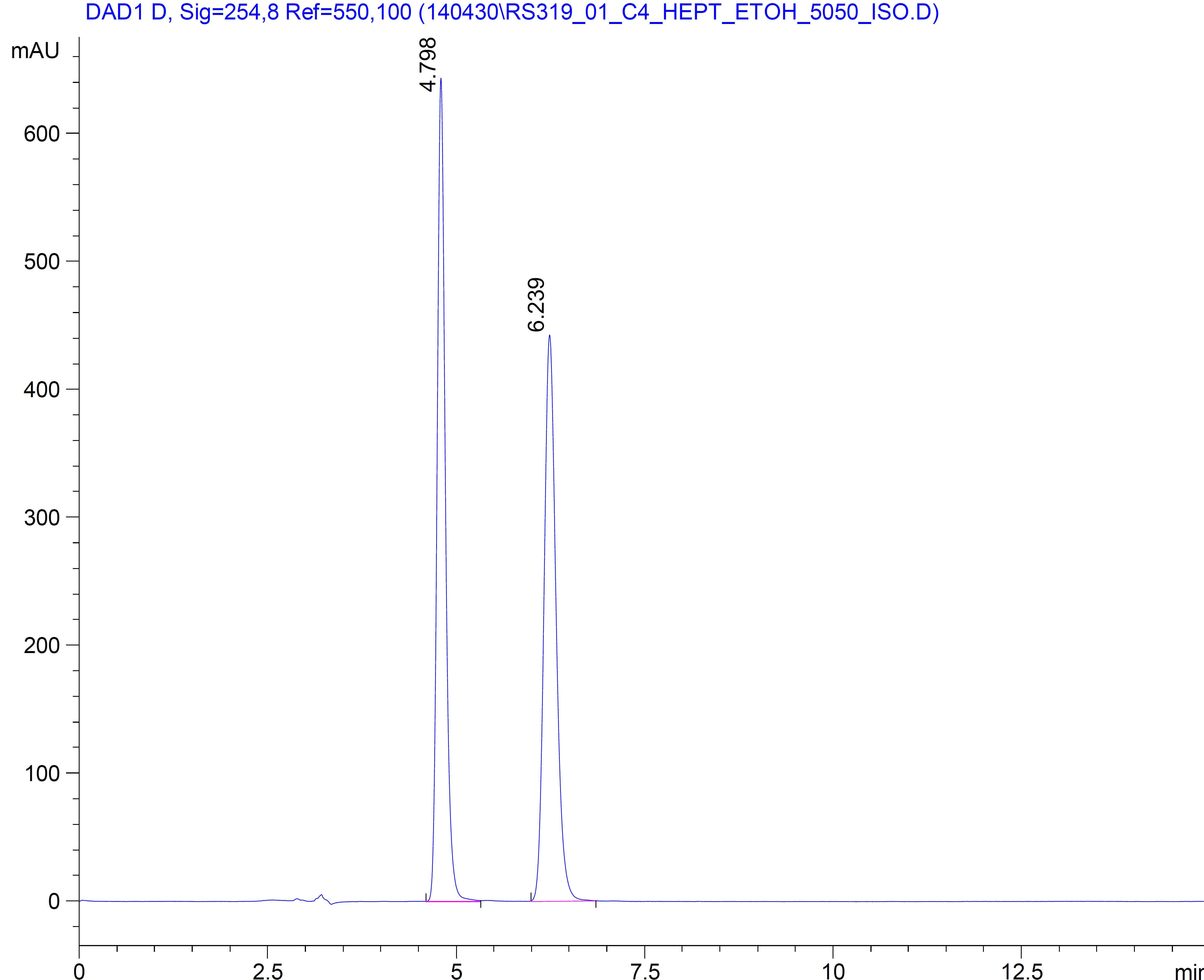


PDA chromatogram for (+)-**4.**

PDA Peak Results:

|  | Retention Time (min) | Area (μV×sec) | % Area | Width (sec) |
| --- | --- | --- | --- | --- |
| 1 | 4.794 | 5.086e3 | 99.56 | 0.129 |
| 2 | 6.212 | 22.284 | 0.44 | 0.167 |


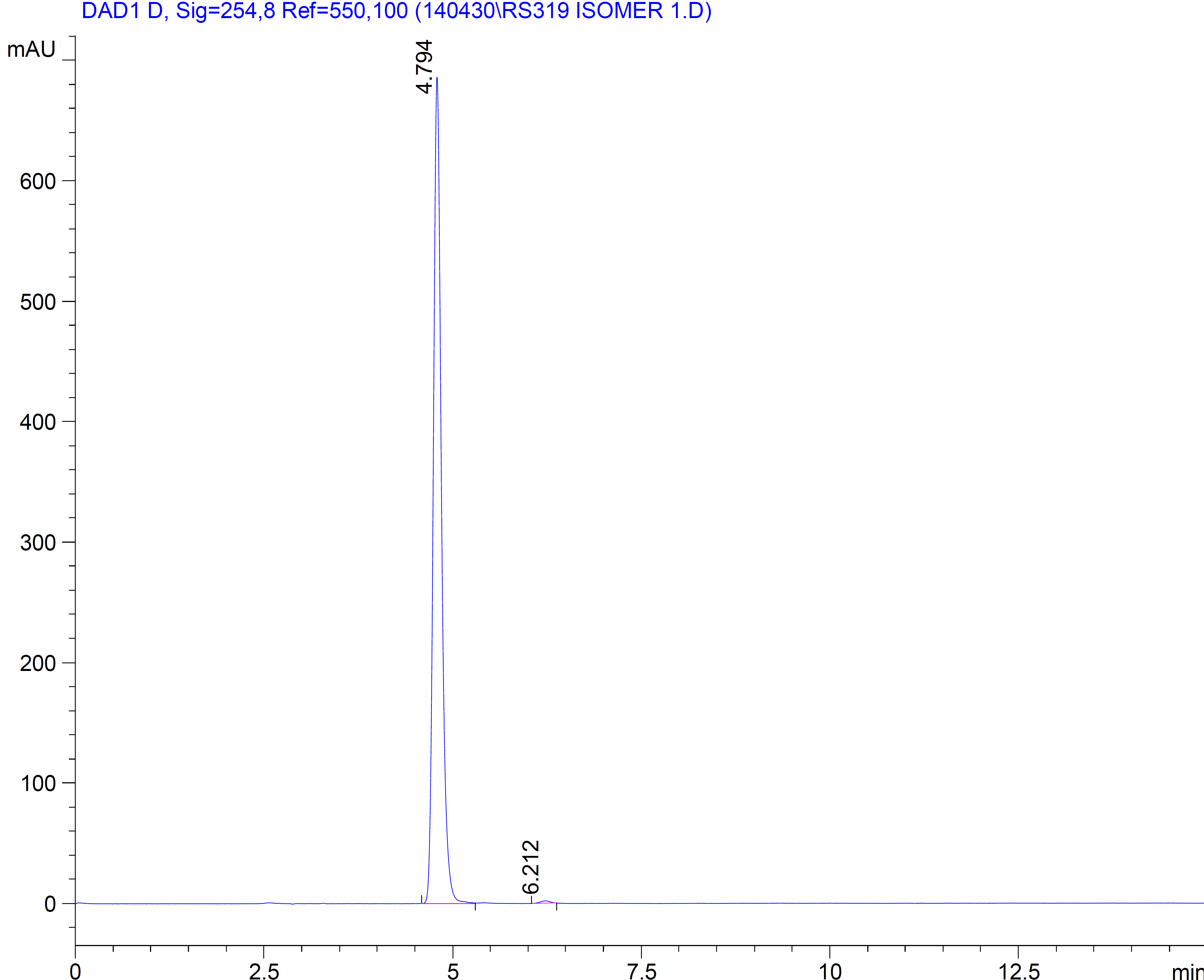


PDA chromatogram for (–)-**4.**

PDA Peak Results:

|  | Retention Time (min) | Area (μV×sec) | % Area | Width (sec) |
| --- | --- | --- | --- | --- |
| 1 | 4.796 | 24.453 | 0.44 | 0.111 |
| 2 | 6.223 | 5.571e3 | 99.56 | 0.175 |


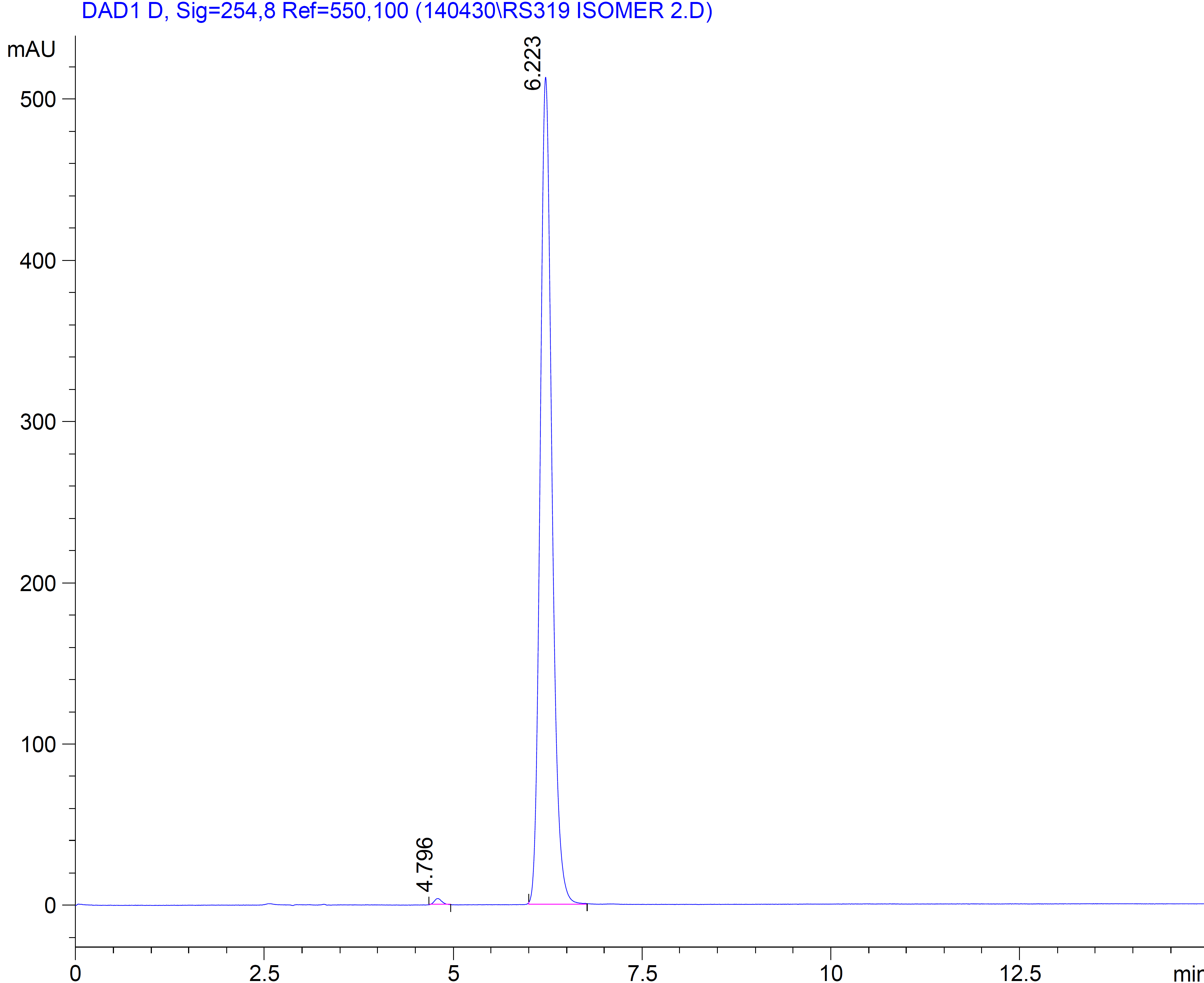


**(1*R*,3a*S*)-1-(2-(3-(Trifluoromethyl)phenyl)benzo[*d*]oxazol-5-yl)tetrahydro-3*H*-pyrrolo[1,2-*c*][1,3,2]oxazaphosphole 1-oxide (*R*_P_-*S*)-9a)**

 ^1^H NMR (500 MHz, CDCl_3_) *δ* **(*R***_P_, ***S*)-9a:** 8.54 (s, 1H)H^13’^, 8.46 (d, *J* = 7.9 Hz, 1H) H^9’^, 8.29 – 8.23 (m, 1H) H^4’^, 7.93 (ddd, *J* = 13.2, 8.4, 1.5 Hz, 1H) H^6’^, 7.83 (d, *J* = 7.7 Hz, 1H) H^11’^, 7.75 – 7.64 (m, 2H) H^7’^+ H^10’^, 4.39 (ddd, *J* = 20.3, 8.8, 6.6 Hz, 1H) H^3α^, 4.20 (dqd, *J* = 8.6, 6.9, 4.4 Hz, 1H) H^6a^, 3.96 (td, *J* = 8.7, 2.4 Hz, 1H) H^3β^, 3.85 – 3.73 (m, 1H) H^6α^, 2.97 (ddt, *J* = 14.5, 10.4, 7.4 Hz, 1H) H^6β^, 2.14 – 1.98 (m, 3H) H^5α^ + H^5β^ + H^4α^, 1.88 – 1.80 (m, 1H) H^4β^; **(*S***_P_, ***S*)-9a’:** 8.55 (s, 1H), 8.47 (d, *J* = 7.6 Hz, 1H), 8.12 (d, *J* = 13.6 Hz, 1H), 7.96 – 7.89 (m, 1H), 7.84 (d, *J* = 8.4 Hz, 1H), 7.78 – 7.71 (m, 2H), 4.79 (ddd, *J* = 15.0, 9.1, 6.4 Hz, 1H), 4.32 (m, 1H), 4.12 (td, *J* = 8.6, 4.1 Hz, 1H), 3.12 – 3.05 (m, 1H), 2.94 – 2.87 (m, 1H), 2.14 – 1.79 (m, 3H), 1.70 – 1.60 (m, 1H).

 **^13^C NMR (126 MHz, CDCl_3_)** *δ* **(*R***_P_, ***S*)-9a:** 162.6 (C^2^’), 153.1 (d, *J* = 3.4 Hz) (C^7a^’), 141.9 (d, *J* = 21.1 Hz) (C^3a^’), 131.7 (q, *J* = 33.0 Hz) (C^12^’), 130.8 (C^10^’), 129.7 (C^9^’), 129.4 (d, *J* = 12.3 Hz) (C^6^’), 128.4 (q, *J* = 3.7 Hz) (C^13^’), 127.9 (d, *J* = 185.1 Hz) (C^5^’), 127.5 (C^8^’), 124.7 (q, *J* = 3.9 Hz) (C^11^’), 124.3 (d, *J* = 11.7 Hz) (C^4^’), 123.7 (q, *J* = 272.6 Hz) (C^14^’), 111.0 (d, *J* = 17.5 Hz) (C^7^’), 69.8 (d, *J* = 2.3 Hz) (C^3^), 63.3 (d, *J* = 7.6 Hz) (C^3a^), 45.4 (C^6^), 30.0 (d, *J* = 3.3 Hz) (C^5^), 27.6 (d, *J* = 1.9 Hz) (C^4^). ^31^P{^1^H} (162 MHz, CDCl_3_) *δ* **(*R*_P_, *S*)-9a:** 38.3; **(*S*_P_, *S*)-9a’:** 32.9.

**Figure 1a:** Stacked ^1^H NMR spectra of (*R*_P_, *S*)-**9a** and (*S*_P_, *S*)-**9a’** in the region between 8.7-7.3 ppm.

Mixture of (*R*_P_, *S*)-**9a** and (*S*_P_, *S*)-**9a’** after 4 h (bottom), after 13 days with formation of species **C** (middle) and clean spectrum of (*R*_P_, *S*)-**9a** (top).

**Figure 1b:** Stacked ^1^H NMR spectra of (*R*_P_, *S*)-**9a** and (*S*_P_, *S*)-**9a’** in the region between 4.9-1.4 ppm.

Mixture of (*R*_P_, *S*)-**9a** and (*S*_P_, *S*)-**9a’** after 4 h (bottom), after 13 days with formation of species **C** (middle) and clean spectrum of (*R*_P_, *S*)-**9a** (top).

**Figure 2:** Stacked ^31^P NMR spectra of (*R*_P_, *S*)-**9a** and (*S*_P_, *S*)-**9a’**.

Mixture of (*R*_P_, *S*)-**9a** and (*S*_P_, *S*)-**9a’** after 4 h (bottom), after 13 days with formation of species **C** (middle) and clean spectrum of (*R*_P_, *S*)-**9a** (top).

**HPLC traces of (*R*_P_)-(+)-4:** The *ee* of (*R*_P_)-(+)-**4** was determined by HPLC analysis. Daicel Chiralcel AD-H column: hexane/*i*-PrOH 90:10, flow rate 1.0 mL/min, 30 ˚C, λ = 220, 254 nm: τ major = 12.0 min., τ minor = 12.7 min.

**SMT022357**

**(*R*_p_)-(+)-4: 83% ee**

**Enantiomer 1**

**Enantiomer 2**

**Figure S1.** HTRF assay. Increase of utrophin protein in iDMD cells

Table S1. HTRF assay. Increase in utrophin protein compared to the untreated cells

|  | **(±)-4** | | | **(+)-4** | | | **(–)-4** | | |
| --- | --- | --- | --- | --- | --- | --- | --- | --- | --- |
|  | 3 uM | 10 uM | 30 uM | 3 uM | 10 uM | 30 uM | 3 uM | 10 uM | 30 uM |
| Increase | 1.09 | 1.27 | 1.20 | 1.22 | 0.92 | 0.46 | 1.05 | 1.22 | 1.22 |

**Figure S2.** Immunofluorescence staining for utrophin on in EDL muscles of mdx mice treated for 5 weeks with 30 mg/kg (+)-**4**, (–)-**4** or vehicle (control).

**
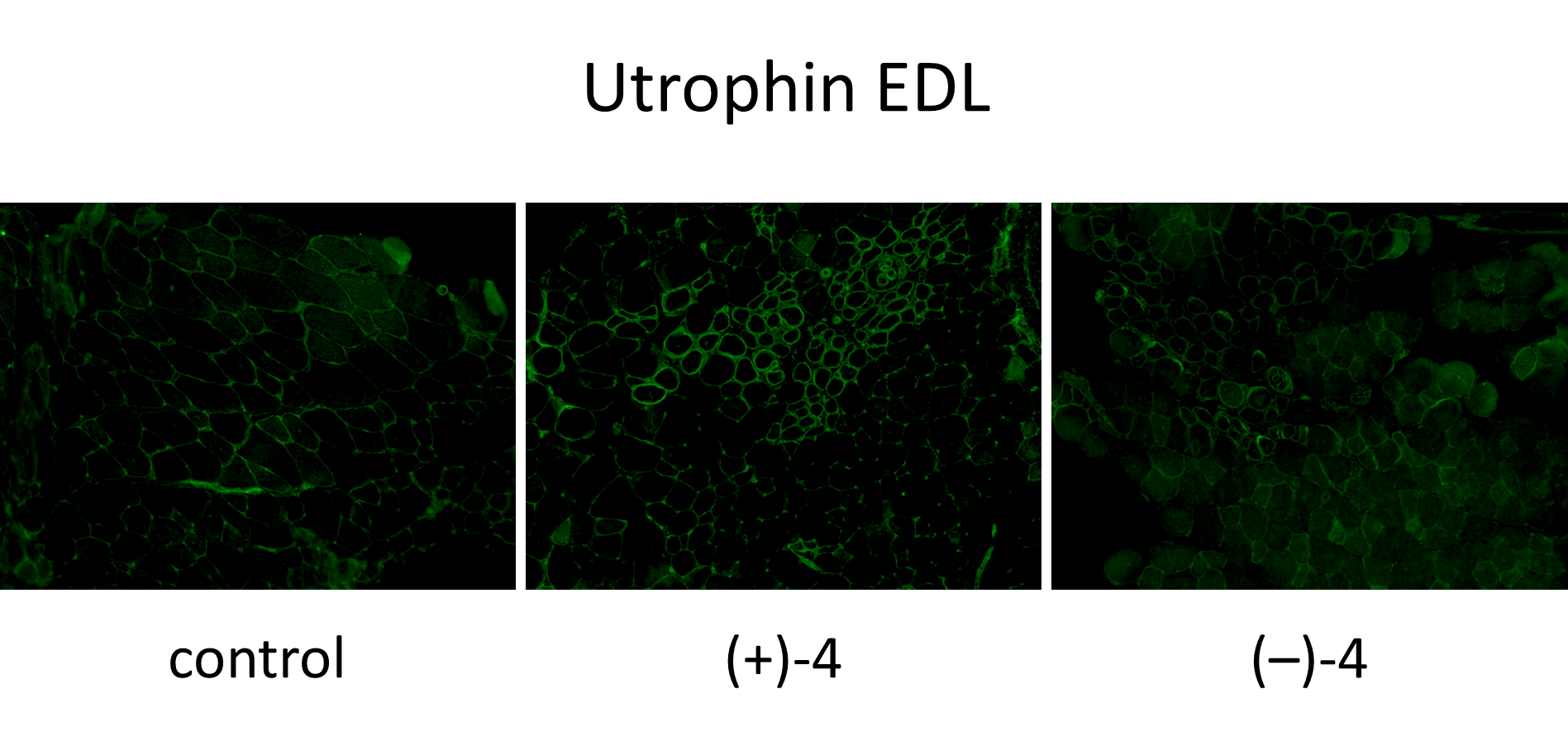
**

**NMR spectra:**

**NOESY experiment for the attribution of the relative stereochemistry of compound (*R*_P_, *S*)*-*9a**


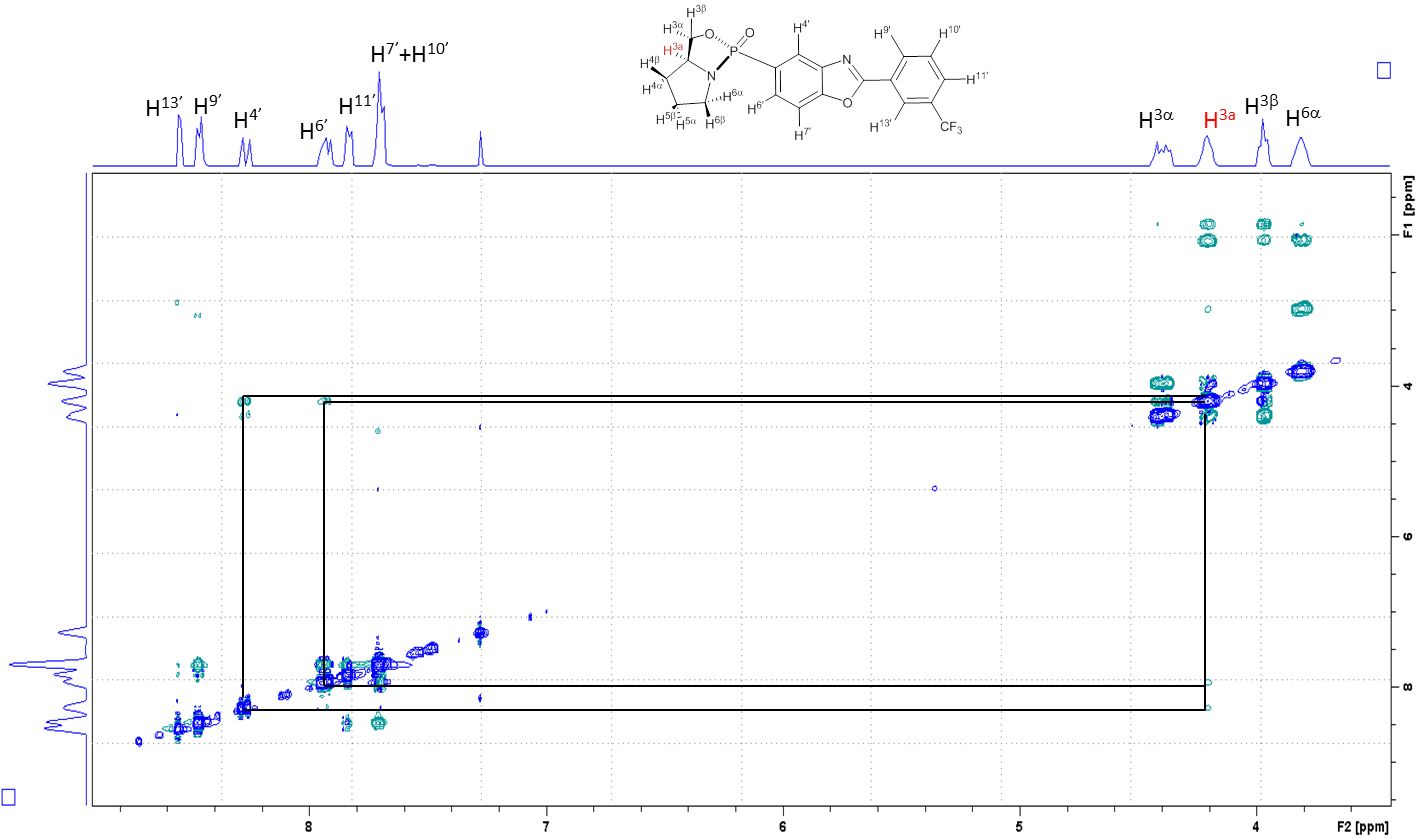

Supplement: Multimedia component 1 [file mmc1.docx]
